# Conserved antigens for enteric vaccines

**DOI:** 10.1016/j.vaccine.2025.126828

**Published:** 2025-03-19

**Authors:** Richard I. Walker

**Affiliations:** PATH, 455 Massachusetts Ave, Suite 1000, Washington, DC, 20001-2621, USA

**Keywords:** *Shigella*, *Campylobacter*, ETEC, Double mutant labile toxin (dmLT), Conserved antigens, Multi-pathogen vaccine

## Abstract

Enterotoxigenic *Escherichia coli* (ETEC), *Shigella*, and *Campylobacter* have been identified as major causes of diarrheal diseases worldwide. In addition to overt disease and death, they are responsible for stunting in children with the risk of lifelong consequences on health and economic opportunities. All three of these bacterial pathogens, which collectively account for approximately 30 % of the cases of diarrheal diseases, are recognized as antimicrobial resistance (AMR) threats. In spite of the dangers these pathogens represent for both children and adults, there is as yet no licensed vaccine available for any of them. Fortunately, much has been accomplished to identify conserved antigens against each of these pathogens so that now relatively simple vaccines have the potential to be developed into multi-pathogen vaccines which could have a major impact on reduction of diarrheal diseases. Conserved antigens may be used even more efficiently if consolidated and expressed on a cellular vector or as part of a conjugate vaccine. A new mucosal adjuvant, double mutant heat-labile toxin (dmLT), has been shown to not only be among the conserved antigens against ETEC, but to also have properties which drive robust mucosal and systemic immune responses for antigens given orally or intramuscularly. Conserved antigens and the strategies for their use such as co-administration with dmLT will be presented in this review.

## Highlights

• Vaccines against ETEC, *Shigella,* and *Campylobacter* are needed.
• Conserved antigens are available for ETEC, *Shigella,* and *Campylobacter.*
• Conserved antigens can facilitate multi-pathogen vaccine strategies.
• dmLT benefits mucosal immunization by oral and parenteral routes.

## 1 The importance of developing a multi-pathogen enteric vaccine

There are few licensed vaccines to significantly reduce the medical and economic impact of diarrheal diseases caused by a myriad of enteric pathogens. The need for an effective and practical vaccine to control major enteric disease pathogens cannot be overstated. Human diseases due to enteric pathogens have been a threat throughout history, yet in many cases the specific causes of these diseases and their pathogenesis has only become known since the 1970s. Even once identified, developing vaccines against these pathogens has been complicated by the number of organisms and their serotypes responsible for enteric diseases. In addition, the challenge of developing effective mucosal immunization strategies has presented a problem for vaccines against enteric pathogens not often encountered with vaccines against systemically infecting pathogens [1]. Today we have a stronger foundation from which to build effective vaccine prevention against enteric pathogens. (See Table 1.)

**Table 1:** Conserved antigens for development of vaccines against major bacterial enteric pathogens.

Enteric Pathogen	Conserved Antigen	Coverage	Status	reference numbers
ETEC	CfaE and CfaEB	Class 5 strains	Phase 2b	42–45
	CssBA	CS6 strains	Phase 2b	46–51
	CFA/I-CS2	Class 5 strains	Preclinical	54
	EatA	ST only and LT only strains; strains with no CFA	Preclinical	55–56, 58
	EtpA	Same as EatA	Preclinical	59
	YghJ	ETEC and other diarrheagenic *E. coli*	Preclinical	60
	dmLT	LT^+^ strains		52, 61–63
	ST	ST^+^ strains	Phase 1	37,76–80
				
Shigella	Ipa B	All species	Phase 1	23–25,27–29
	Ipa C	All species	Phase 1	24, 31, 33
	PSSP1	All species	Preclinical	30
				
Campylobacter	N-glycan heptasaccharide	All species	Preclinical	100–105
	FlaA	All species	Preclinical	81, 87, 90
	JlpA	All species	Preclinical	81, 91–92
	CTB (Por A)	All species + cholera toxin	Preclinical	94–99

Bacterial pathogens account for much of the enteric disease burden in low- and middle-income countries (LMICs) where *Campylobacter, Shigella*, and enterotoxigenic *Escherichia coli* (ETEC) are found to account for one third of the global diarrheal disease burden [[2], [3], [4]]. Approximately one third of the remaining cases are due to rotavirus and the remaining third are due to other causes. In addition to morbidity and mortality due to acute diarrheal disease, infections with these three pathogens are associated with both physical and intellectual stunting in children as well as other long-term sequelae of enteric infection, including reactive arthritis, Guillain-Barre Syndrome, and an increased risk of mortality due to other infectious diseases in stunted children [[5], [6], [7]]. These three pathogens are also designated as antimicrobial resistance (AMR) threats by the World Health Organization (WHO) and other public health stakeholders [8]. Therefore, vaccine development should be prioritized for all three pathogens [9,10] before we no longer have effective antibiotic treatment options available to us. In addition, all three pathogens were recognized for their longer-term negative health effects on infants and young children in LMICs [11] and all three remain the primary bacterial causes of Traveler's diarrhea [12]. Although some gains have been made in reducing cases by improving sanitation, access to clean water, and availability of rotavirus vaccines, these gains are threatened by rapidly accelerating climate changes that are expected to exacerbate the conditions associated with an increased risk of enteric infections [[13], [14], [15]].

## 2 Conserved and consolidated antigens for development of practical multi-pathogen vaccines

The most effective way to reduce the impact of enteric diseases is to focus future efforts towards development of multi-pathogen vaccines rather than standalone products against each pathogen. Part of the challenge of developing effective multi-pathogen vaccines against enteric pathogens is that, in addition to there being multiple species associated with disease episodes, there are often multiple serotypes associated with individual clinically important pathogens. This is important because addition of multiple standalone single pathogen vaccines, each containing a number of antigenic components, to already crowded vaccination schedules in LMICs is not a desirable or acceptable goal [[16], [17], [18]]. Thus, multi-pathogen vaccines providing broad coverage for the major pathogens for which promising vaccine antigens are available, are necessary to better insure global uptake and beneficial public health impact on enteric disease-associated morbidity, mortality, and antibiotic use.

Development of combined pathogen vaccines is often approached by identifying the major clinical serotypes or species of each pathogen responsible for clinical disease and then focusing vaccine development efforts towards those antigens. For example, to protect against most *Shigella* using the sero-specific O-polysaccharide (OPS) antigens, requires three OPS serotypes for *S. flexneri* (2a, 3a, and 6) and one serotype for *S. sonnei.* To combine these four OPS antigens with the similar number of antigens to provide broad coverage against ETEC, for example, while certainly possible would be a more challenging goal. This approach has been simplified through antigen consolidation, through use of attenuated *Shigella* strains engineered to express the major fimbrial and toxin antigens of ETEC [19]. Consolidation by using one pathogen as a platform to also express the antigens of another can reduce the total number of antigens necessary to prepare the vaccine. In addition, safety concerns related to use of attenuated strains in infants and young children may also be addressable since preclinical studies indicate that even inactivated constructs expressing the consolidated antigens of other pathogens can be immunogenic and protective in animal models [20]. This may be simpler to achieve with cellular vaccines expressing heterologous antigens for oral administration compared to subunit vaccine candidates for parenteral administration. In addition, development of an oral vaccine can simplify achievement of a mucosal immune responses to be most effective against enteric pathogens and also avoid further use of needles. In the future, exploitation of broadly conserved antigens can further minimize the number of individual components in oral whole-cell or injected subunit vaccines and thereby should reduce costs and difficulty of antigen acquisition and manufacture.

Recent research has identified conserved antigens [21] to potentially provide sufficient coverage for much needed multi-pathogen vaccines. Such antigens have been identified for *Shigella,* ETEC, and *Campylobacter.* In spite of this accomplishment, only a few of these antigens have as yet been developed into vaccine candidates. This progress and the possibilities for use of conserved antigens against these three pathogens are summarized below.

### 2.1 Conserved antigens: *Shigella*

The two most common *Shigella* species are *S. flexneri* and *S. sonnei* which are transmitted through the fecal-oral route or through ingestion of contaminated food and water. Once ingested, they can cause an invasive disease of the colonic epithelium effected by its type III secretion system (T3SS), a needle-like molecular syringe anchored in the bacterial cell wall [22]. The T3SS consists of conserved proteins such as the invasion plasmid antigens IpaB, IpaC, and IpaD, which can offer protection against disease [23,24]. The other major protective antigens of Shigellae are serotype-specific O-polysaccharides of the cell wall.

Both conserved and sero-specific antigens can contribute to protection against *Shigella*. For example, serum samples from volunteers immunized with killed, attenuated, and wild-type *S. flexneri* 2a were assessed in a core *Shigella* proteome microarray [25]. In this study, antibodies against the invasion plasmid antigens IpaA, IpaB, IpaC, IpaD, and IpaH were associated with clinical protection. In a more recent proteomic and lipopolysaccharide array analysis, antibody levels against both *S. sonnei* lipopolysaccharides (LPS) and IpaB were associated with a reduced risk of developing shigellosis and a reduced disease severity score following infection with the 53G strain of *S. sonnei* [26].

In support of the microarray findings, T3SS proteins involved in cellular invasion and spread have shown broad protection in animals. For example, IpaB and IpaD have been used to protect mice against lethal challenges from multiple serotypes of *Shigella* [[27], [28], [29]]. A less studied conserved *Shigella* protein which may also be important for protection is a surface polypeptide (IcsP) found to be a prominent cross-protective moiety, pan-*Shigella* surface protein 1 (PSSP-1), common to over 300 *Shigella* strains tested [30]. PSSP-1 adjuvanted by mucosal administration of cholera toxin (CT) or dmLT induces broad protection in mice against experimental challenge with strains belonging to all major species and serotypes of *Shigella* [30]. The conserved T3SS proteins and the PSSP-1 have led to novel approaches to make *Shigella* vaccines.

#### 2.1.1 Invaplex_AR-Detox_

The most studied approach using T3SS antigens is the *Shigella* vaccine candidate Invaplex_AR-Detox_ [24,31]. Invaplex_AR-Detox_ is a subunit *Shigella* vaccine consisting of *S. flexneri* 2a and *S. sonnei* LPS complexed with the broadly conserved IpaB and IpaC proteins*.* The LPS utilized in the Invaplex_AR-Detox_ was purified from a msbB *Shigella* mutant that expresses under-acylated and therefore detoxified Lipid A. This innovation is not only important for safety during intramuscular administration, but also allows the full immunogenic potential of the LPS to be realized for the immunologic benefit of the vaccine recipient. The recombinant Ipa proteins are produced in a unique, tag-free method prior to assembly in a complex with LPS. The vaccine has been engineered to induce immune responses capable of protecting against multiple serotypes and is therefore considered to be a pan-*Shigella* vaccine. Transition to the under-acylated Lipid A has widened the vaccine-associated safety window to permit parenteral administration by reducing, but not completely abrogating, the “danger” signal associated with endotoxin, resulting in a safe yet highly immunogenic vaccine [32].

A Phase 1 trial [33] conducted in 60 adults in the United States studied three different intramuscularly injected dose levels (2.5, 10, and 25 μg) of Invaplex_AR-Detox_ given in a three-dose course. Although the complete vaccine will contain both *S. flexneri* 2a LPS and *S. sonnei* LPS, this was a monovalent prototype formulation containing, in addition to the Ipa proteins, LPS from *S. flexneri* 2a. Invaplex_AR-Detox_ was safe and well-tolerated when administered intramuscularly to adults at all dose levels tested. All three dose levels of Invaplex_AR-Detox_ were highly immunogenic, inducing strong systemic antibody responses to all *Shigella* antigens contained within the vaccine. In addition to robust systemic responses, the monovalent prototype Invaplex_AR- Detox_ vaccine elicited significant mucosal IgG and IgA responses with an average of 93 % of vaccinated subjects demonstrating a mucosal antibody response across all dose levels. Importantly, all doses induced significant levels of functional serum bactericidal antibodies against *S. flexneri* serotypes that were comparable or above those achieved by experimental infection and peaked after the first dose and persisted for over one year.

#### 2.1.2 OPS-IpaB conjugate

Although OPS conjugates have been one of the major approaches to developing vaccines against *Shigella*, the carrier proteins used have not been relevant to the pathogen. A recent report describes a novel *Shigella* OPS conjugate vaccine that uses IpaB as the carrier protein and as a protective antigen [34]. As seen with Invaplex, the *S. flexneri* 2a OPS-IpaB conjugate given parenterally was immunogenic and protected mice against challenge with *S. flexneri* 2a or *S. sonnei*. When protection against *S. flexneri* 2a was determined following immunization with OPS-IpaB, OPS-CRM, or IpaB alone, there was a trend towards higher protective efficacy with the OPS-IpaB than either of the other two preparations. This suggests that higher protection was attributable to the combination of the OPS and IpaB. The importance of the IpaB in the conjugate is further indicated by the 56 % protection following challenge with *S. sonnei* obtained in mice immunized with OPS-IpaB. This conclusion is supported by the similar protection (44 %) seen with IpaB alone. In contrast, no protection was observed in animals given the OPS-CRM before challenge (11 % survival).

It is presently unknown if OPS to both *S. flexneri* and *S. sonnei* need to be included in vaccines also including conserved proteins. Further work with Invaplex or the OPS-IpaB conjugate could show that immunity to T3SS proteins may need to be augmented with OPS immunity only for *S. sonnei*. This species is an encapsulated organism whereas *S. flexneri* and other *Shigella* species are not [35]. The presence of this capsule polysaccharide renders the organism less invasive in vitro as a consequence of the capsule-mediated shielding of the T3SS. Thus, in *S. sonnei* the Ipa proteins at the tip of the T3SS necessary for invasion, could be less accessible to immune responses. The IpaB and IpaC in the *S. sonnei* preparation may be sufficient to protect mice against *S. flexneri* 2a where the T3SS is more accessible, but the LPS of *S. sonnei* may become the more important protective antigen against this species where the Ipa proteins are not as accessible [36].

#### 2.1.3 ShigETEC

Two oral whole-cell approaches, one attenuated and one inactivated, have also exploited conserved antigens to protect against *Shigella.* ShigETEC is *S. flexneri* 2a attenuated by the deletion of the T3SS and is engineered to not express any LPS-O antigens through a targeted deletion of the *rfbF* gene [37]. Removal of the serotype-determining O-antigen component of LPS exposes surface proteins and facilitates broad protection against heterologous *Shigella* strains. Immunization of mice three times at two-week intervals provided 100 % protection of animals infected intranasally with lethal doses of heterologous wild-type *Shigella* strains: *S. flexneri* 6 or *S. sonnei*. The expression of the ETEC proteins LTB and a detoxified ST (generated by a single amino acid change) by this vaccine strain aims to address ETEC for a broader coverage of diarrheal pathogens.

The ShigETEC live oral vaccine was well-tolerated in a first-in-human study [38], wherein 48 subjects were randomized and sequentially enrolled in four ascending dose groups to receive a single oral dose of the ShigETEC vaccine ranging from 1 × 10^9^ colony forming units (CFU) of vaccine or a placebo, to 2 × 10^11^ CFU per dose group. The study showed that the oral ShigETEC vaccine was well tolerated at doses up to 5 × 10^10^ CFU/dose at up to four doses at 3-day intervals with only minor and transient reactogenicity noted. While the antibody responses to the ST were more modest compared to those against *Shigella* and LTB, it represents the first reported successful generation of anti-ST serum antibodies with an oral live attenuated vaccine.

#### 2.1.4 Shigella truncated mutant (STM)

The other oral whole-cell approach builds on an antigen strategy involving pan-*Shigella* surface proteins [30], in that it involves the unmasking of surface antigens such as PSSP-1 on the whole cell by limiting the length of polysaccharide chains synthesized. This is the *Shigella* Truncated Mutant (STM), which utilizes genetically modified (*∆wzy*) chemically inactivated bacteria retaining only one repeating unit of O antigen chain on the bacterial surface [39]. This process broadened protective immunogenicity. The STM combined with dmLT induced potent antibody responses to the outer membrane protein PSSP-1 and T3SS proteins IpaB and IpaC. Intranasal immunization of mice with the vaccine preparation elicited cross-protective immunity against *S. flexneri* 2a, *S. flexneri* 3a, *S. flexneri* 6, and *S. sonnei.* Thus, *S. flexneri* 2a *∆wzy* represents a promising candidate strain for a universal *Shigella* vaccine.

Both of these novel approaches using orally administered whole cells, increase exposure of conserved outer membrane proteins, and could effectively provide a broad coverage *Shigella* vaccine with a single or few cell types. Both may provide exposure of the PSSP-1 protein, but only the STM would also retain and present potentially protective proteins of the T3SS and some sero-specific immunity. In addition, there may be other novel proteins on the surface of these “unmasked” constructs that could contribute to protection [25]. These two cellular vaccine candidates both offer the possibility for antigen consolidation for coverage of heterologous antigens as has already been done with ShigETEC.

### 2.2 Conserved antigens: ETEC

ETEC colonize the small intestine where they employ plasmid-encoded fimbrial colonization factors (CFs) or coli surface antigens (CS) to bind enterocytes in the upper small intestine. This is followed by production of heat-stable (ST) and/or heat-labile (LT) enterotoxins that stimulate the release of fluid from the intestinal epithelium, resulting in watery diarrheal illness [40,41]. ETEC surface-expressed CFs and LT are critical virulence factors*.*

#### 2.2.1 Canonical antigens

Instead of using the whole antigenic structure of fimbria, as was done with some other ETEC vaccine candidates, two prototype CF-derived subunit vaccine antigens (CssBA and CfaE) covering the most prevalent ETEC adhesins (CS6 and CFA/I, respectively) have been developed [42,43]*.* Both antigens are protective against diarrhea in a non-human primate (NHP) model [44,45]. Moreover, the CS6-derived antigen, CssBA, formulated with dmLT was safe and immunogenic when administered intramuscularly, and elicited intestinal mucosal responses in a Phase 1 human trial [[46], [47], [48]]*.* Intradermal administration of CfaE and mLT (single mutant of labile toxin (LT_R192G_)) was safe and immunogenic, and reduced the incidence and severity of diarrhea in volunteers in a controlled human infection model (CHIM) with the CFA/I ETEC strain H10407 [47,48]*.* Optimized from the prototype vaccine CfaE, CfaEB protected against diarrhea in the NHP model following challenges with a homologous CFA/I-expressing ETEC strain, as well as a strain expressing CS14 [49]. Notably, antibodies to CfaE showed a broad class 5 ETEC CF antigen response [50,51], suggesting that vaccines containing CfaE or CfaEB can confer protection over heterologous class 5 ETEC strains*.* In the above studies, the LT component (dmLT or mLT) has adjuvanted the serum and mucosal responses to the CF-based antigen and has induced robust anti-LT responses, which are independently associated with a reduction in ETEC attributable diarrhea risk and disease severity [52,53]. Taken together, these data support the development of a multivalent ETEC vaccine consisting of CssBA, CfaEB, and dmLT. This vaccine candidate could be co-mixed with other subunit vaccines, or the two adhesins could be expressed on vaccine vectors to provide multi-pathogen coverage.

An alternate approach to the use of the CfaEB antigen has been to construct hybrid fimbriae containing the major subunits of CFA/I as well as CS2. These were expressed in high amounts on a recombinant *E. coli* strain. In addition, oral immunization with formalin-killed bacteria expressing hybrid II fimbriae gave rise to strong serum as well as intestinal antibody responses against both CFA/I and CS2 in preclinical studies [54].

#### 2.2.2 Non-canonical proteins

Studies of ETEC pathogenesis have enabled the discovery and characterization of novel non-canonical antigens [[55], [56], [57]] which could lead to new conserved antigens for ETEC vaccines. Vaccination of mice with EtpA or EatA effectively reduces the intestinal colonization of mice by ETEC [58]. The inclusion of these non-canonical antigens in ETEC vaccine candidates could complement or broaden the protection afforded by CF-based antigens. For example, data emerging from these studies suggest that a combination of CS6 and EtpA could afford coverage for more than 80 % of ETEC [58,59]. These non-canonical antigens are also shared across other strains of diarrheagenic *E. coli* beside ETEC; thus, they may provide broader protection in vaccines that contain them. If the non-canonical antigen YghJ is added, for example, it could also bring coverage for uropathogenic *E. coli* and strains associated with neonatal sepsis [60]. As discussed in reference 21, EatA shares a ∼ 75 % amino acid identity with SepA secreted by some strains of *Shigella*. Whether this could be exploited for multi-pathogen coverage remains to be determined.

#### 2.2.3 Antitoxin

Anti-toxin activity against the heat labile toxin (LT) of ETEC can be provided safely by cholera toxin B subunit (CTB) or dmLT. The dmLT has also been shown to induce anti-LT antibody responses in human volunteers and to induce better LT-toxin neutralization antibody responses than B-subunit toxoid preparations of LT (LTB) [52,61]*.* Field and CHIM study data indicate that inducing anti-LT immunity can be an immune marker for reduced risk of ETEC illness, particularly from ETEC strains expressing only LT toxin, and that anti-LT antibodies can reduce the severity of ETEC diarrhea in subjects infected with LT/ST-producing strains [51,62].

Immunity against LT may also be of benefit for reducing the long-term consequences of infection with ETEC, which have been linked repeatedly to poorly understood sequelae including enteropathy, malnutrition, and growth impairment. While the cellular actions of ETEC enterotoxins leading to diarrhea are well-established, their potential contribution to subsequent pathology is unclear. Recently Sheikh et al. [63] reported that LT treatment of intestinal epithelial cells significantly disrupted the absorptive microvillus architecture and altered transport of essential nutrients. In addition, challenge of neonatal mice with LT-producing ETEC recapitulated the architectural derangement of the brush border. Maternal vaccination with LT prevented brush border disruption in ETEC-challenged neonatal mice. These findings highlight the impacts of ETEC enterotoxins beyond acute diarrheal illness and may inform approaches to mitigate and prevent major sequelae, including malnutrition, that impact millions of young children. In addition, intoxication of the small intestine by LT is also thought to make it more susceptible to colonization and disease caused by other bacterial enteric pathogens [64]. Consequently, neutralization of LT effects may also serve to reduce the risk of infection and disease in infants, children, and adults.

In addition to offering antitoxin protection against acute and long-term effects of ETEC infection, LT is un**i**que in that it is not only a potent antitoxin component for a future ETEC vaccine, but it is also an effective mucosal adjuvant [65,66]. In fact, new adjuvant data from 6- to 11-month-old Bangladeshi infants, a difficult population in which to achieve robust immunity following the oral administration of vaccines, show that dmLT improves the frequency and the magnitude of the mucosal immune response to ETEC antigens following immunization with a killed whole-cell vaccine, ETVAX [67]. Furthermore, in Bangladeshi infants, the kinetics of the mucosal immune response was accelerated by the inclusion of dmLT in the vaccine. In addition, these studies in infants in Bangladesh have also shown that the dmLT adjuvant can improve the mucosal antibody response to both protein and polysaccharide antigens, and that the use of dmLT was particularly important for ETEC vaccine immune responses in 6- to 11-month-old infants, as fractional doses of the vaccine were required to improve its tolerability [68]. This immune enhancement was most apparent after the first vaccine dose. This observation was particularly encouraging as earlier studies with inactivated whole cell oral cholera vaccines in this age-group have consistently exhibited poor immunogenicity and efficacy [69]. The results with ETVAX suggest that dmLT may help overcome the difficulty in effectively immunizing this age-group at high risk for enteric diseases, like ETEC and cholera. In earlier work, the live attenuated ETEC candidate vaccine ACE527 provided significant protection in a Phase 2b challenge study only when the vaccine was co-administered with dmLT [70].

Vaccines against enteric diseases in LMICs could be more readily administered and possibly more effective in infants and children comprising the target population if they could be given parenterally. Co-administration of a mucosal adjuvant like dmLT with antigens given parenterally is a paradigm shift for more effective immunization with subunit enteric vaccines. Few adjuvants when combined with vaccine antigens can achieve strong mucosal immunity, but among those that are capable are the bacterial ADP-ribosylating enterotoxins such as cholera toxin (CT) and dmLT. Given intradermally or intramuscularly they cannot only increase the level of immune response, but also can direct an immune response to the intestinal mucosa [[71], [72], [73], [74], [75]]. These findings indicate that it is not the route of administration but the adjuvant that drives the localization of the immune response.

ST-ETEC is the primary cause of pediatric diarrhea in LMICs [76], yet ST has only recently been developed as a vaccine component. Recent work has shown that the ST molecule can be detoxified without losing neutralizing properties, be made immunogenic by linking to a protein, and avoid cross reaction with human guanylin/uroguanylin [77]. It has been expressed as a toxoid on an attenuated *Shigella* in ShigETEC and used successfully in a Phase 1 clinical study [38]. It is also a toxoid component in MecVax, a preclinical ETEC vaccine candidate where antigenic components of seven adhesins, and ST and LT are attached to a protein backbone for parenteral administration [78,79]. Passive immunization studies in piglets have shown protection against disease in animals immunized with MecVax [80].

### 2.3 Conserved antigens: *Campylobacter*

*Campylobacter* is a motile invasive pathogen of the colon. It causes tissue damage and ultimately disease manifestations [81]. *Campylobacter* may be responsible for more than 160 million cases of gastroenteritis each year [82] and is linked to growth stunting of infants living under conditions of poor sanitation and hygiene [83]. Recent studies in infant rhesus macaques have illustrated that both these acute and long-term consequences of infection with this organism can be reduced by vaccination with a killed whole-cell *Campylobacter* vaccine [84]. In spite of the importance of a vaccine against *Campylobacter,* this goal has not been as vigorously pursued as have vaccines against ETEC and *Shigella.* Nevertheless, conserved antigens have been identified that may offer broad protection against *Campylobacter* strains.

#### 2.3.1 Conserved *Campylobacter* proteins

The antigenic properties of several recombinant *Campylobacter* proteins have been evaluated [81]. This work indicates that the conserved proteins FlaA and JlpA could be broadly protective antigens against *Campylobacter.* Flagellin is the immunodominant antigen recognized during *Campylobacter* infection and development of antibodies against flagellin correlates with protection against disease [[85], [86], [87], [88], [89]]. Early CHIM studies demonstrated that volunteers challenged with *C. jejuni* strain 81–176 developed a robust immune response against the flagella. In addition, the magnitude of the anti-flagellin response correlated with protection against disease [90]. To generate a recombinant flagellin-based vaccine, the conserved region of FlaA from *C. coli* VC167 was fused with the maltose binding protein (MBP) of *E. coli*. The vaccine (rFlaA-MBP) was formulated with mLT and tested for immunological response and protective efficacy in a mouse colonization model [87]. Encouraging results were obtained: an intestinal secretory IgA response and protection against heterologous colonization and disease by *C. jejuni* 81–176 were established when the rFlaA-MBP was adjuvanted with mLT.

Another protein of *Campylobacter*, JlpA, was present in all *C. jejuni* strains and thus, like FlaA, is highly conserved [91]. JlpA is a surface adhesin glycosylated *Campylobacter* lipoprotein that is immunogenic during natural human infection. Recently *Lactococcus lactis* was used to express functionally active JlpA protein on the surface. This construct substantially improved the antigen-specific local immune responses in the intestine along with significant immune-protection against enteric colonization by *C. jejuni* in chickens [92].

#### 2.3.2 Cross-reactive CTB protein

In addition to offering some protection against cholera and ETEC enterotoxins (CTB shares over 83 % sequence identity with LT) [93], CTB also cross reacts with a surface protein of *C. jejuni****.*** Many *Campylobacter* strains possess a 53 kDa protein which reacts with CT antibody by immunoblotting. It was identified as the major outer membrane protein PorA [[94], [95], [96]]. A recombinant PorA fused to glutathione-S-transferase co-administered with mLT was protective against three unrelated serotype strains used to challenge an adult mouse intestinal colonization model [97]. Further, orally immunizing mice with CT protected against colonization with different serotypes of *Campylobacter* and this cross protection was mediated by CTB [95]. Thus, inclusion of the relatively easy to manufacture CTB could facilitate preparation of a vaccine against *C. jejuni*.

Clinical evidence for a possible contribution of CTB against *Campylobacter* was obtained from a study of travelers to Mexico and Guatemala who received an inactivated whole-cell ETEC vaccine candidate which contained CTB. The vaccine not only reduced the incidence and severity of ETEC, but subjects with serum IgA titers to CTB had a lower risk of infection with ETEC and *C. jejuni/coli* [98,99].

#### 2.3.3 N-glycan Heptasaccharide

An N-glycan that is a common and invariable feature among *C. jejuni* isolates has been described [100,101]. The N-glycan was used to construct glycoconjugate vaccines combining the conserved *C. jejuni* with a protein carrier, GlycoTag, or fused to the *E. coli* lipopolysaccharide-core. Vaccination of chickens with either vaccine preparation containing the N-glycan showed up to a 10-log reduction in *C. jejuni* colonization [[102], [103], [104]]. Immunization of mice with this construct also induced functional opsonizing anti-*Campylobacter* antibodies that enhanced the killing of *Campylobacter* in vitro [105].

## 3 Conclusions and future directions

Conserved antigens have now been identified (Table 1) that can make multi-pathogen enteric vaccine preparations not only practical but more obtainable. One of these antigens, dmLT, also serves as an effective mucosal adjuvant whether used orally or parenterally, facilitating options for multi-pathogen vaccines. A review of the data now available clearly indicates that using conserved and consolidated antigens can bring much-needed multi-pathogen vaccines closer to reality than they have ever been before. This coupled with a dual market for protection against these pathogens for travelers as well as children in LMICs, should further drive the incentive to produce vaccines suggested by recent advances described in this report.

Each year, approximately 1.7 billion global diarrheal episodes occur and nearly 500,000 children under five years old die from severe, dehydrating diarrhea and dysentery, and millions more are hospitalized, mostly in LMICs [106,107]. There has never been a better opportunity to produce much needed multi-pathogen vaccines against major bacterial enteric pathogens with relatively few individual vaccine components. This minimization may be improved by consolidation, where heterologous antigens are expressed on cellular vectors. The challenge today is less the science, and more the poor climate to support and guide attainment of the opportunities now on the horizon. Success will require managing a triangle of science, donor support, and industrial partnerships necessary to bring a product from research to realization among target populations in a relatively near-term time frame. Many manufacturers have been reluctant to pursue an enteric vaccine product and donor support in this field has been erratic. For example, the US Army/10.13039/100000005Department of Defense, previously a leader in the enteric vaccine field, has deprioritized its internal program to develop vaccines as a means to control enteric infections, and strategy refreshes at several key donor organizations have led to a major reduction of support for enteric vaccine development, particularly for multi-pathogen products. Somehow these losses must be overcome. It is time to exploit the scientific advances available to bring about a major medical advance, now made more possible through development of conserved and consolidated antigens. It is time to give multi-pathogen enteric vaccine candidates the priority they deserve.

## CRediT authorship contribution statement

**Richard I. Walker:** Writing – review & editing, Writing – original draft, Project administration, Investigation, Formal analysis, Conceptualization.

## Declaration of competing interest

The authors declare that they have no known competing financial interests or personal relationships that could have appeared to influence the work reported in this paper.

## Data Availability

No data was used for the research described in the article.
